# A Biochar-Based Route for Environmentally Friendly Controlled Release of Nitrogen: Urea-Loaded Biochar and Bentonite Composite

**DOI:** 10.1038/s41598-019-46065-3

**Published:** 2019-07-02

**Authors:** Xiangrong Liu, Jiayuan Liao, Haixing Song, Yong Yang, Chunyun Guan, Zhenhua Zhang

**Affiliations:** 1 Southern Regional Collaborative Innovation Center for Grain and Oil Crops in China, College of Resources and Environmental Sciences, Hunan Agricultural University, Changsha, 410128 China; 2 Hunan Qidong Country Tobacco Company, Hengyang, 421600 China; 3 National Engineering Laboratory on Soil and Fertilizer Resources Efficient Utilization, Changsha, 410128 China

**Keywords:** Sustainability, Composites

## Abstract

Biochar-based fertilizers have attracted increased attention, because biochar can improve the soil fertility, promote plant growth and crop yield. However, biochar-based controlled release nitrogen fertilizers (BCRNFs) still face problems because of the high cost, inefficient production technology, instability of nitrides, and the challenge associated with the controlled release of nutrients. In this study, we hydrothermally synthesised novel BCRNFs using urea-loaded biochar, bentonite and polyvinyl alcohol for controlled release of nutrients. Scanning electron microscopy and gas adsorption were conducted to identify the urea-loading and storage of bentonite in the inner pores of the biochar particles. X-ray diffraction, Fourier transform infrared spectroscopic and X-ray photoelectron spectroscopic studies demonstrated that strengthening the interactions among biochar, urea, and bentonite, helps control the moisture diffusion and penetration of bentonite, thereby leading to nutrient retention. The BCRNF showed significantly improved nutrient release characteristic compared with that of a mixture of biochar and urea. This urea-bentonite composite loaded with urea provides control over the release of nutrients stored in the biochar. BCRNF, especially those produced hydrothermally, can have potential applications in sustainable food security and green agriculture.

## Introduction

Nitrogen fertilizer is essential for maintaining the soil fertility and plant growth, but nitrate leaching, ammonia volatilisation, and nitrous oxide emissions cause high N losses, resulting in low efficiency of N utilisation, high economic costs, and environmental pollution^1–4^. Previous studies have paid much attention to use polymers as coating materials for achieving controlled- release of urea^5^. Although the coating materials can retard the nutrient release rates, the cost, nondegradability, and nonrenewability of such coating materials lead to negative environmental and economic impacts^6^. An increasing number of studies have focused on bio-based coating materials for developing controlled- release nitrogen fertilizers (CRNFs) to improve the N utilisation efficiency and reduce the environmental impact^7,8^. However, the cost of bio-based coating materials, complicated production process, and the catastrophic release when coating is damaged with cracks, transportation and degradation restrict further development of CRNFs^6,8^.

Biochar is considered as a potentially attractive, sustainable, and green support and coating material for CRNFs, because of the cost effectiveness, environmental friendliness, recyclability, renewability, and desirable physical-chemical properties for industrial scale application^3,9,10^. Biochar is a carbon-rich, porous substance with multiple-functional groups that could increase nutrient retention, increase water retention, and enhance soil fertility, and thereby promote plant growth and crop productivity^9,11–13^. However, application of high amounts of biochar, > 10 t ha^−1^, is not agronomically and economically feasible, because N, P, and K are not present in substantial quantities^12,14–16^. In some approaches, fertilizers (mainly P fertilizers) are combined with biochar to obtain biochar-based fertilizers via direct mixing, co-pyrolysis^17^, compounding^18^, co-composting^19^, sorbing nutrients from a solution^20,21^, and microwave synthesis^22^. Hydrothermal synthesis is a simple, reactive, and highly efficient approach to produce nanoparticles, carbonates, self-assembled compounds, and silicates which depends on the solubility of minerals and materials in hot water under high pressure in sealed vessels^23–26^. However, very few biochar-based CRNFs have been reported, and none of the studies attempted to combine biochar with nitrogen fertilizers by the hydrothermal synthesis to produce CRNFs, because of the thermal decomposition of nitrides.

Raw materials to produce biochar, including plant residues, wood chips, organic wastes, and poultry manure, are widely available^27^. Oilseed rape straws are a cheap, abundant available, renewable, and biodegradable agricultural waste. Oilseed rape is one of the world’s major oilseed crops, and worldwide rapeseed production reached 73 million metric tons in 2017^28^. Bentonite is a natural silicate mineral containing montmorillonite with a lamellar structure. It is considered a good substrate for CRNFs, because of its water-retention property, swelling capacity, valuable CEC, thermal stability, slow-release property, and reactive OH groups on its surface^29–31^. Polyvinyl alcohols (PVA), an emulsifier and coating agent, are synthetic polymers widely used in industrial, commercial, medical, and food applications because of they are cheap, biodegradable, and nontoxic^32^.

In this work, we synthesized biochar-based controlled-release nitrogen fertilizers (BCRNFs) with water retention via a hydrothermal method and characterised their physicochemical and morphological properties. We analyzed the controlled release of nutrients for BCRNFs as well as their water-holding ratio (WH%) and water-retention ratio (WR%) in soil. Results reveal that BCRNFs produced using hydrothermal strategies may be agriculturally effective and economically feasible, rendering them attractive for widespread use for sustainable agriculture and green revolution.

## Results

### Physicochemical and morphological characteristics

The main physicochemical properties of biochar and produced fertilizers were listed in Table S1. After hydrothermal synthesis, the N contents increased from 1.24 g/kg for biochar to 38.2 g/kg for BCRNF and 75.1 g/kg for biochar nitrogen fertilizer (BNF). The specific surface area (SSA) of biochar decreased from 23.84 m^2^/g to 0.62 m^2^/g for BCRNF.

### X-ray diffraction (XRD) studies

Figure S1 shows the XRD patterns of samples reflections at 2θ = 28.39° and 40.65°associated with the disordered stacking of micro-graphites, consistent with the lower crystallinity of biochar. The characteristic reflection of urea also appeared at 2θ = 22.61°, 24.95°, 29.65°, 31.85°, 35.91°, 37.33°, 45.73°, 49.84°, and 55.24° in the XRD patterns of BNF-2 and BNF-4 indicating that biochar was effectively filled and loaded with CO(NH_2_)_2_. The characteristic reflections of CO(NH_2_)_2_ weakened after the hydrothermal synthesis, probably because of the filling and loading of CO(NH_2_)_2_ into the porous structure of biochar, its reaction with the acidic functional groups, or binding to the specfic adsorption sites on the surface of biochar, which led to a decrease in the crystallinity of CO(NH_2_)_2_. The biochar reflections could still observed at 2θ = 31.1°, 40.65°, and 43.5°, indicating that its structure remained stable. Compared with the pattern of biochar, the characteristic reflection of BNF-2, BNF-4, and BBNF at 2θ = 28.39° weakened or disappeared, suggesting that the crystal structure changed or weak graft polymerization of biochar and CO(NH_2_)_2_ occurred during the hydrothermal process. Additionally, no obvious peaks are observed in the XRD patterns of BCRNF indicating that PVA had coated biochar, because PVA has no crystallographic planes.

### Fourier transform infrared spectroscopy (FTIR) and X-ray photoelectron spectroscopy (XPS)

**Figure 1:**
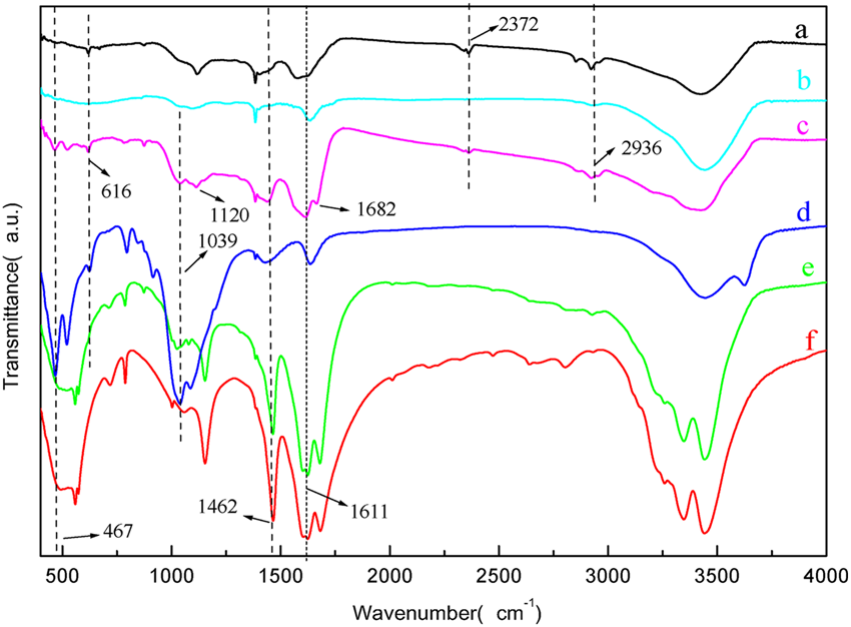
FTIR spectra of biochar (**a**), PVA (**b**), BCRNF (**c**), bentonite (**d**), BNF-2 (**e**), and urea (**f**).

**Figure 2:**
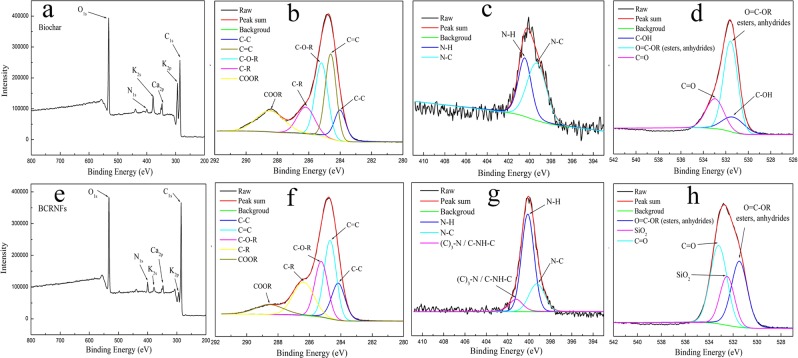
XPS survey scans of biochar and BCRNF on indium foil. Analysis of the biochar surface modification induced by hydrothermal synthesis. XPS scanning spectra of biochar (**a**) and BCRNF (**e**) show three major peaks of carbon, nitrogen, and oxygen. XPS high resolution survey scan of C1s (**b**,**f**), N1s (**c**,**g**) and O1s (**d**,**h**) region of biochar and BCRNF.

FTIR spectrum (Fig. 1) of BNF-2 (e) showed features similar to that of urea (f), the peaks associated with N-H stretching (3450 cm^−1^, 1462 cm^−1^) and C=O stretching of the acyl bond (1611 cm^−1^), indicate that urea covered up the features of biochar (a). Biochar showed a characteristic adsorption band at 1383 cm^−1^ attributed to C=O symmetric stretching vibration of COO− groups. Comparison of the FTIR spectrum of BCRNF (c) with the FTIR spectra of biochar (a), PVA (b), bentonite (d), BNFs-2 (e), and urea (f) in Fig. 2, indicated that the characteristic absorption band of bentonite at 3620 cm^−1^ (stretching vibration of Al-OH and Mg-OH) disappeared, while the bands at 1039 cm^−1^ (Si-O stretching vibration) and 467 cm^−1^ (Si-O-Si bending vibration) weakened in the BCRNF spectrum, implying that the -OH groups of bentonite reacted with biochar and PVA, and bentonite chemically bonded with the polymer chains of PVA^29^. In the FTIR spectrum of BCRNF, the absorption bands at 2372 and 2936 cm^−1^ were corresponded to the stretching vibration of aromatic C=C and C-H out-of-plane in biochar, while the absorption band at 3432 cm^−1^ was ascribed to OH stretching and overlapped with these of N-H groups. The characteristic peaks at 1120, 1462 and 1682 cm^−1^ were ascribed to the C-NH-C asymmetric stretching vibrations, amine III C–NH bond, and CH2–CO–NH– bending vibrations, respectively^33,34^.

Furthermore, XPS was performed to characterise the surface elemental composition and chemical bonds of the BCRNF and the results are compared with that of biochar. The distinct peaks at 285.8, 400.6, and 531.4 eV presented in the full XPS spectra (Fig. 2a,e) are assigned to C1s, N1s, and O1s, respectively. Biochar and BCRNF were found to be mainly composed of C, N, and O as confirmed by the XPS assay, which corresponds with the elemental analysis results. In the expanded XPS spectra, the C1s spectra of biochar and BCRNF (Fig. 2b,f) exhibited five peaks at 284.2, 284.7, 285.3, 286.5, and 288.5 eV, attributed to C-C, C=C, C-O-R, C-R and COOR (R=H, N), respectively^35^. The XPS characteristic peaks of PVA overlapped with those of biochar. The N1s peaks of biochar at 399.3 and 400.5 eV shown in Fig. 2c are assigned to nitrogen in the form of N-C and N-H groups. The three peaks in the N1s spectrum of BCRNF (Fig. 2g): N-C at 399.3 eV, N-H at 400.5 eV, and amine III C-NH ((C)_3_-N) at 401.2 eV, are in agreement with the FTIR spectral analysis^36^. The O1s spectra (Fig. 2d,h) exhibit peaks at 531.3, 531.6, 532.5, and 533.0 eV, associated with C-OH, COOR (esters, anhydrides), SiO_2_ and C=O groups, indicating the presence of bentonite. The integral areas of peaks were normalized and the percentages of the chemical bonding of C1s, N1s and O1s are shown in Table S2.

### Scanning electron microscopy (SEM) and X-ray energy dispersive spectroscopy (EDS)

**Figure 3:**
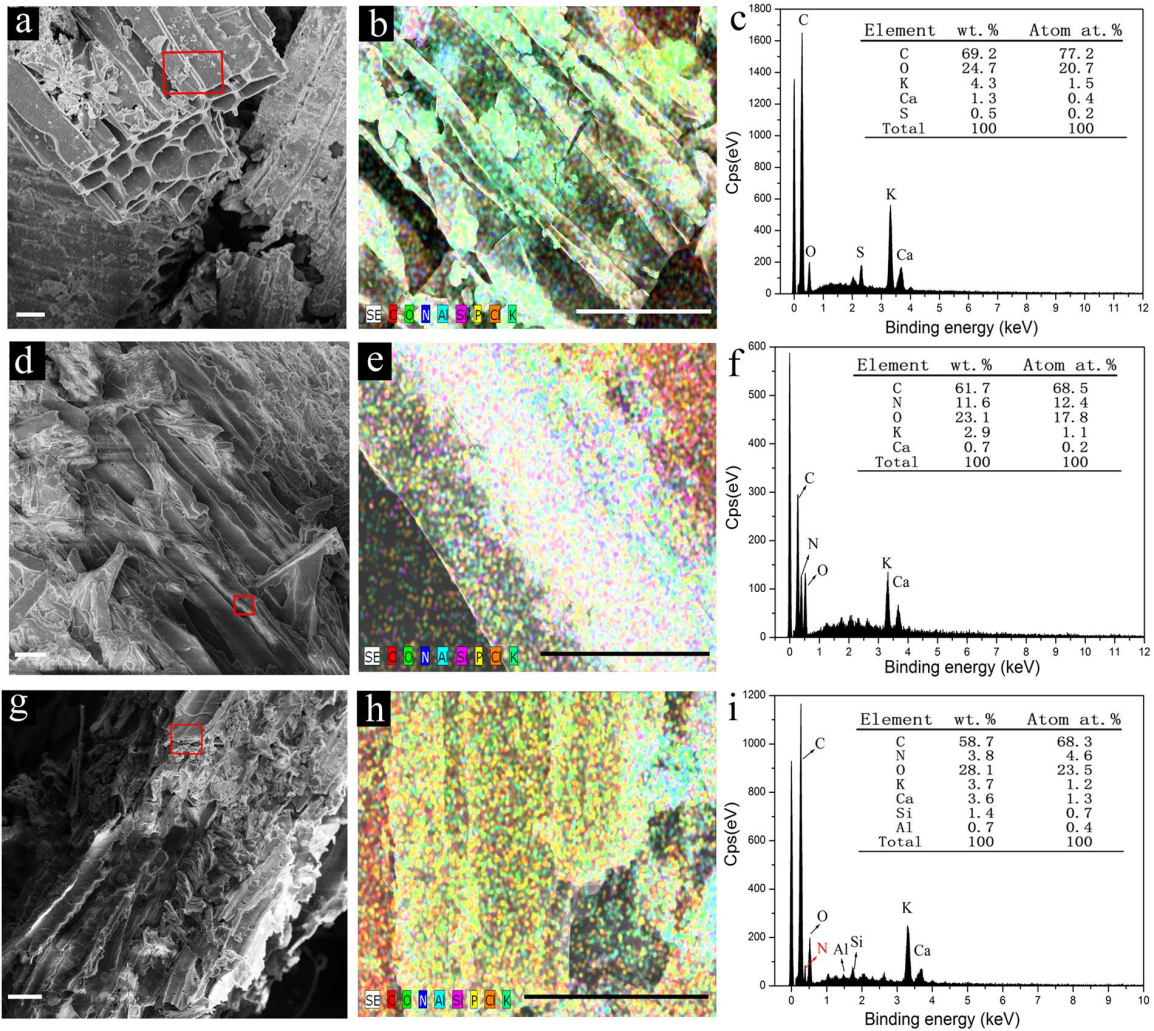
Scanning electron micrograph (SEM) of Biochar (**a**), BNF-2 (**d**), and BCRNF (**g**) (scale bar, 20 µm). The map overlay of the red selected region of Biochar (**a**), BNF-2 (**d**), and BCRNF (**g**) showing the heterogeneous distribution of C, N, O, Al, Si, P, Cl, and K in (**b**), (**e**), (**h**), respectively (scale bar, 20 µm). The corresponding X-ray energy dispersive spectra (EDS) of the red selected region of Biochar (**c**), BNF-2 (**f**), and BCRNF (**i**) are shown in the right column.

SEM is largely used for characterizing the surface morphology and composition of biochars (composites), especially for detecting macro-pores and channels of biochar. Biochar exhibited a porous structure with a smooth, tight, and micro-tube bundle morphology (Fig. 3a)^37^. The macro-pores and channels in the highly porous structure of biochar allow it to favourably store nutrients and soil microorganisms, retain nutrients, and reserve water during irrigation or rain periods. Some urea particles filled the pores and exposed cavities of biochar, and crystallized on the surface of biochar unevenly, in which many fissures, cracks, and collapsed locations could be observed (Fig. 3b). BCRNF (Fig. 3c) exhibited a coarse and undulating surface, attributed to the graft polymerization of PVA with biochar and bentonite, as well as to the insertion of urea and bentonite into the cavities and channels of biochar. This polymeric network structure could control the permeation of water into the porous biochar and the release of nutrients into the soil. Therefore, a high water-potential gradient could be generated between the inner and outer parts of the pore channels, when biochar is in a moist environment^38^.

Surface element analysis was conducted simultaneously with the SEM at the same surface locations using EDS, to perform rapid semiquantitative analysis of the elemental composition. The map overlay of the red selected region of biochar, BNF-2, and BCRNF shows a heterogeneous distribution of carbon, oxygen, nitrogen, potassium, calcium, silicon, and aluminium (Fig. 3b,e,h). SEM elemental mapping images of C, N, O, Al, Si, P, Cl and K for the sectioned region of biochar, BNF-2, BCRNF were shown in Figs S2–S4, respectively. SEM elemental mapping revealed the presence of nitrogen, silicon, and aluminium on the surfaces of biochar, BNF-2, and BCRNF, indicating the graft polymerization of PVA with biochar, urea and bentonite. EDS analysis of biochar revealed a low N content and the largest percentages of carbon and oxygen, indicating that biochar was rich in carbon and contained traces of nitrogen. The O/C ratio of BNF-2 is found to be ~0.374, close to the value obtained for the original biochar (24.7/69.2 ≈ 0.357), but much less than that of the ratio O/C of urea, suggesting interaction between urea and biochar.

### SSA and pore size distribution

**Figure 4:**
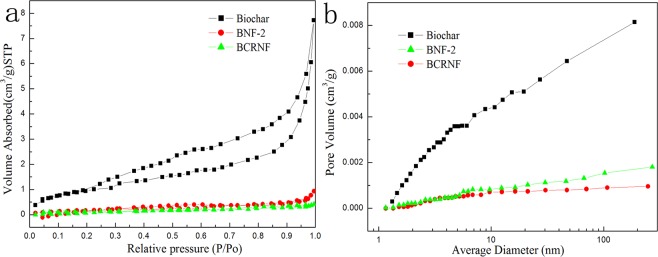
Nitrogen Adsorption-desorption isotherms of Biochar, BNF-2, and BCRNF (**a**). Pore size distribution of Biochar, BNF-2, and BCRNF (**b**).

The N_2_ adsorption−desorption isotherms (Fig. 4a) of biochar, BNF-2, and BCRNF with small hysteresis loops extending from P/P0 = 0.20 to 0.99 have type II characteristic (according to the IUPAC classification), suggesting the existence of macropores in the biochar^39^. Pore size distribution curves of biochar, BNF-2, and BCRNF (Figs 4b and S5) indicate that the pore size, especially that of the macropores, decreased dramatically after the hydrothermal synthesis. Hydrothermal processing led to ~98% reduction in SSA, likely due to the reduced size and number of pores, indicating that urea particles, bentonite, and PVA had been inserted into the micropores and mesopores in the channel and filled the macropores and exposed cavities of the biochar.

### Controlled-release behavior of BNF-2, BBNF, and BCRNF in water and soil

**Figure 5:**
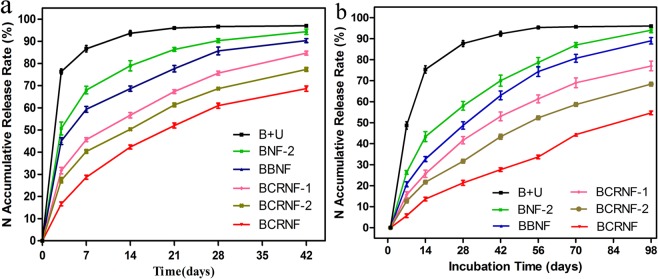
N release behaviour of B + U (40 g of biochar mixture with 2 g of urea prepared by grinding), BNF-2, BBNF, and BCRNF in water (**a**). N release behavior of urea, BNF-2, BBNF, and BCRNF in soil (**b**).

Simple water incubation experiments were carried out to characterise the nutrient release rate and the cumulative amount of nutrient released from BCRNF. B + U (a ground mixture biochar (40 g) and urea (2 g)) showed the fastest release rate, with almost 94% of nitrogen released into water after 14 days (Fig. 5a). However, the rates of release from BNF-2, BBNF, BCRNF-1, BCRNF-2, and BCRNF, were retarded, with the cumulative amounts of N released into water only reaching 90.4, 86.7, 75.6, 68.6, and 61.3% within 28 days, respectively. The nutrient release plots clearly show the controlled release behaviour of BCRNF. The N release profiles of BNF-2, BBNF, and BCRNF follow analogous parabolic diffusion models, indicating that the release process involves a combination of dissolution, adsorption and diffusion processes^40^. According to the SEM results (Fig. 3), urea particles on the surface or within the pores and cavities of biochar are released into water with their dissolution and diffusion.

The agricultural application of control release fertilizers usually involves their burial in soils. To objectively evaluate the application prospects in agriculture and horticulture, we studied the controlled-release characteristics of BCRNF in soil. The release profile of B + U indicates that it readily dissolved in water and was released easily into the soil (Fig. 5b). The release rates of N from BNF-2, BBNF, BCRNF-1, BCRNF-2, and BCRNF (Fig. 5b) were slower than that from B + U, with the respective cumulative N released being 94.8, 89.3, 77.2, 68.3, and 54.6% within 98 days, respectively, and the release was not complete at the end of the experiment. The nitrogen release from BNF-2 was much lower than that of B + U, owing to the adsorption and coating of biochar with urea in the former. Compared to cumulative release of N from BNF-2, BBNF showed limited release of nutrients into soil, perhaps due to the adsorption of bentonite, and hydrogen bonding and electrostatic interactions of the N-C=O and oxygen-containing functional groups, all of which may hinder the release rate of N. Finally, complete release of N from BCRNF was dramatically prolonged, which is likely due to the network structure of polymeric PVA and the graft polymerization of PVA with biochar and bentonite.

### Water-holding and water-retention capacities of soil with BNF-2, BBNF, and BCRNF

Efficiently managing soil moisture and reducing water consumption are serious challenges for farmers. Herein, the WH% and WR% capacities of soil were determined for soils treated with BNF-2, BBNF, and BCRNF. WH% of soils treated with BBNF and BCRNF increased compared to those of the untreated soil (Fig. S6a). The largest WH% of soil samples (untreated soil, and soil with 2% of BNF-2, BBNF and BCRNF) are found to be 40.4, 53.7, 72.7, and 69.8%, respectively. Therefore, the BCRNF could improve the soil moisture and reduce water loss in agriculture. BNF-2 had a comparatively less impact on the WH% of soil. Meanwhile, BBNF markedly affected the WH% of soil, because the water adsorbtion capacity of bentonite is far higher than that of biochar. For the same reason, the WH% of BCRNF was lower than that of BBNF, likely due to the water absorption performance of bentonite.

Control soils that were not treated with biochar additives lost all absorbed water after 18 days (Fig. S6b), whereas the moisture of soils with BNF-2 was prolonged to 27 days. The soils with BBNF and BCRNF still retained 9.6, and 5.8% of the soil moisture on the 30th day, respectively. The water loss rates of soil mixed with BCRNF were obviously lower than those treated with others, indicating that BCRNF have a more pronounced effect on the WR% than BNF-2, and BBNF. BCRNF showed excellent water absorbency, because of the hydrophilic functional groups and porous structure of bentonite and biochar, the grafting of PVA with bentonite and biochar, and the polymer network of PVA on the surface of biochar. The absorbed water of BCRNF could be gradually released into the soil when the moisture content of soil reduced; further, the nutrients incorporated into water could also be gradually released into the soil, and subsequently taken up by crops.

### Morphologies of post-release B + U and BCRNF

**Figure 6:**
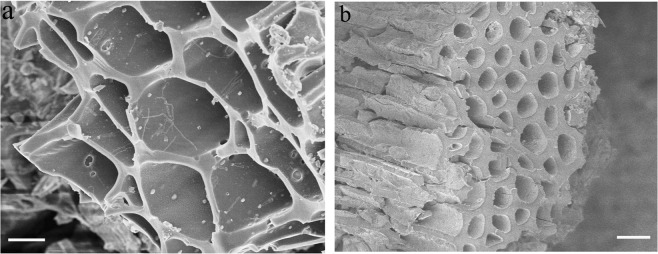
SEM of B + U (**a**) and BCRNF (**b**) after nutrient release.

After the release of nutrients into water, the microstructures of B + U and BCRNF are characterized by SEM. B + U shows smooth and tight surface morphology (Fig. 6a), and micro-holes were observed on biochar. However, BCRNF displayed an undulating and coarse surface (Fig. 6b). The nanoscale particles (many nanoscale rough bulges on the wall of the holes) are scattered in the holes of biochar, which is attributed to the graft polymerization of PVA with bentonite and biochar as well as to the introduction of bentonite.

### Relationships among the amount of biochar, bentonite, PVA, and nutrient release characteristics

**Figure 7:**
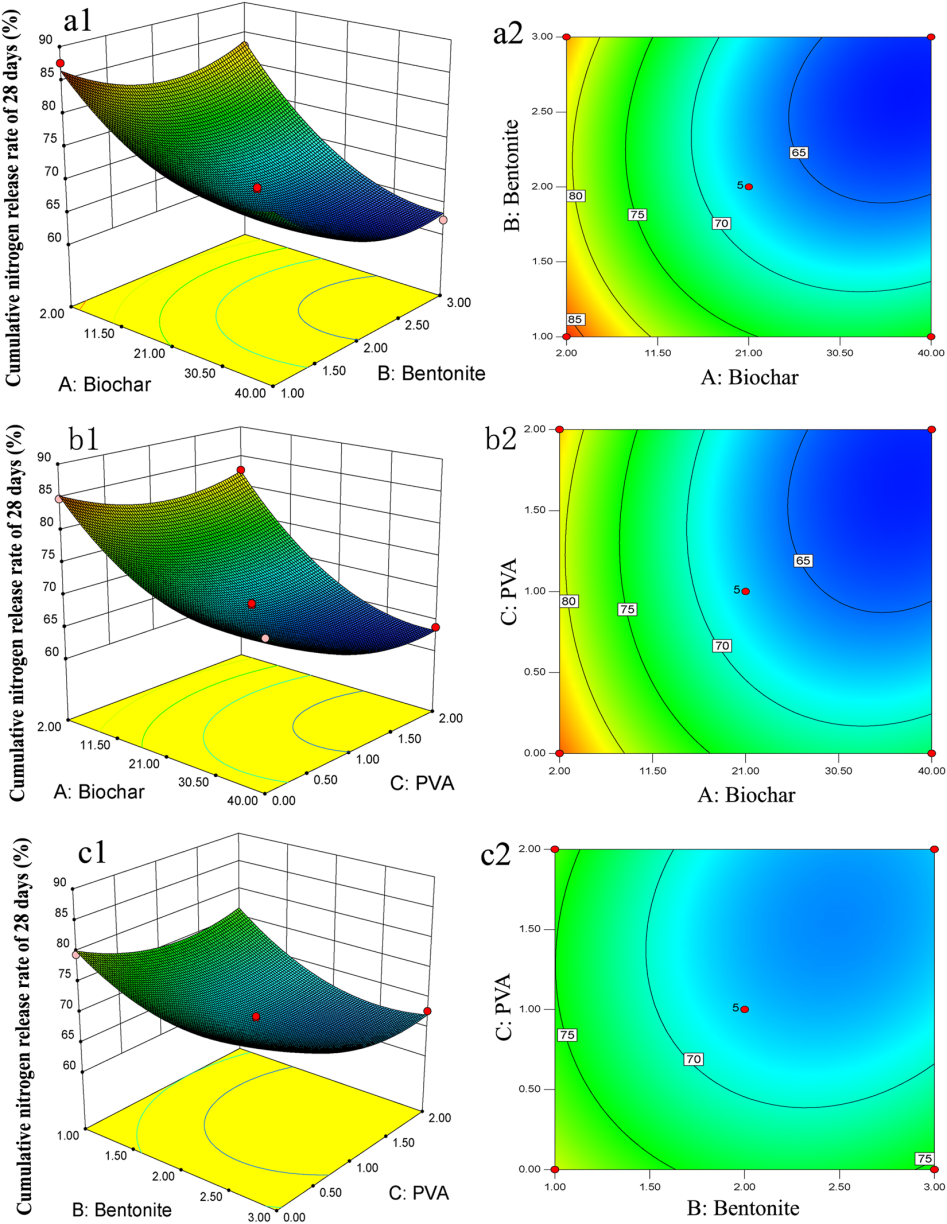
Relationship between cumulative nitrogen release of biochar- based fertilizers over 28 days and the amount of biochar, bentonite, and PVA. Three-dimensional modelling (**a1**) and interaction contour maps (**a2**) of biochar (A) and bentonite (B) on cumulative nitrogen release over 28 days. Three-dimensional modeling (**b1**) and interaction contour maps (**b2**) of factors biochar (A) and PVA (C) on cumulative nitrogen release rate of 28 days. Three-dimensional modelling (**c1**) and interaction contour maps (**c2**) of bentonite (B) and PVA (C) on cumulative nitrogen release over 28 days. Significance at 0.05 probability level.

The results of controlled-release behaviour indicated that increasing the amount of biochar, bentonite, and PVA significantly improved the release longevity of BCRNF. However, high biochar, bentonite, and PVA content could decrease the nutrient content and increase the cost; therefore, the amount of biochar, bentonite, and PVA should be appropriately controlled. The effects of the amount of biochar, bentonite, and PVA on the design array for nutrient release characteristics (Y) were evaluated (Fig. 7). The cumulative nitrogen release rate over 28 days (Y) response value was fitted with a quadratic model (R2 = 0.9899). The resulting models relating the amount of biochar (A), bentonite (B), and PVA (C) were determined as follows:

$${\rm{Y}}=104.43-0.73{\rm{A}}-17.30{\rm{B}}-5.12{\rm{C}}-0.11{\rm{AB}}-0.08{\rm{AC}}-1.23{\rm{BC}}+0.01{{\rm{A}}}^{2}+4.33{{\rm{B}}}^{2}+3.18{{\rm{C}}}^{2}$$

The correlation coefficients R^2^ (0.9899) and adjusted R^2^ (0.9770) were evaluated. The variance analysis was used to evaluate the above model equations. The polynomial model was extremely significant in this experiment, as the p-value for Y is <0.0001 (P > 0.05). This model is suitable for analysing and predicting the influence of the amounts of biochar, bentonite, and PVA on the release behaviours of BCRNF. Based on factors such as the release characteristics and the agricultural expenditure, the optimum composition is 32.4 g of biochar, 2.36 g of bentonite, and 1.33 g of PVA.

## Discussion

### Temperature of pyrolysis and hydrothermal synthesis

Details of discussion of pyrolysis temperature are given in the Supporting Information. According to previous studies and analysis, the appropriate pyrolysis temperature for biomass is 400 °C to obtain higher yield and quality of biochar products with high nutrient retention properties.

Hydrothermal synthesis has been proven to be an efficient strategy for both the development of new compounds with specific physical properties and the systematic physicochemical investigation of intricate multicomponent systems, partly because new compounds can be prepared at vapour pressures near their melting points. Therefore, a temperature for hydrothermal synthesis of BCRNF near the melting points of urea (132−135 °C) is suitable. Urea is unstable at high temperatures and deaminates into biuret when heated to 150~160 °C. Consequently, the temperature near 135 °C for hydrothermal synthesis seems to be optimal.

### Interactions among urea, bentonite, PVA, and biochar

It is difficult to accurately determine the interaction between urea, PVA, and biochar because of the many co-existing COOH, C-OH, C-O, C-N, N-C=O, and C-N=O functional groups in biochar, and N-C=O and C-N-H functional groups in urea. From the binding energies corresponding to the electron configuration of the electrons within the atoms of C and N obtained by XPS, it is impossible to distinguish the carbon and nitrogen energy spectra of urea or biochar itself or to determine the interaction between urea and biochar. Fortunately, XRD (Fig. S1) indicated that CO(NH_2_)_2_ probably reacted with acidic functional groups, or bound to species sites on the surface of biochar. The FTIR (Fig. 1) and XPS (Fig. 2) spectra proved the complexation between C-NH-C and CH_2_-CO-NH groups in BCRNF, and bentonite bonded with the polymer chains of biochar and PVA. Moreover, the FTIR study indicated that the nutrient sorption by biochar can be attributed to the chemisorption of polar and nonpolar compounds, including hydrophobic bonding^41^, π-π electron donor-acceptor interactions resulting from fused aromatic carbon structures^42^, and weak unconventional H-bonds^43^. The binding to and within biochar might take a number of forms, such as ligand exchange, dissolved multidentate ligand, C-π-cation interaction, acid-base reactions with COOH, C-OH, C-O, and C-N functional groups on the biochar surface, coordination of deprotonated OH or COOH functional groups, and reactions of O-containing groups with multivalent Al^3+^, Ca^2+^, and NH_4_^+^ cations^44,45^. For example, NH_4_^+^ bound to biochar, owing to van der Waals adsorption^46^, electrostatic attraction between NH_4_^+^ and negatively charged surfaces^47^, binding of NH_4_^+^ to cationic species on the surface of biochar^48^, reaction of NH_4_^+^ with acidic functional groups to form amides and amines^49^, and π-π electron donor-acceptor interactions^50^. In the FTIR and XPS spectra, the newly formed C-NH-C and CH_2_-CO-NH complex agree with C-π-cation interaction and π-π electron donor-acceptor interactions. The results of XRD, FTIR, and SEM-EDS combined with the results of previous researches provided evidences that urea particles entered the inner pores of biochar and the newly formed complex could associate with biochar to form an organic complex.

### Controlled-release mechanisms of BCRNF

Peng *et al*.^22^ reported the cumulative N release into water at 47.3% within 7 days and 69.8% within 28 days from biochar-based slow-release nitrogen fertilizer; the values are higher than those observed in our study (28.67% and 61.3%). Wen *et al*.^29^ also developed a slow-release nitrogen fertilizer based on urea incorporated into a composite of sodium alginate, acrylic acid, acrylamide and bentonite via microwave irradiation, and reported cumulative N release in soil of 56.1% within 30 days, which is higher (by ~20%)than those achieved in our study.

**Figure 8:**
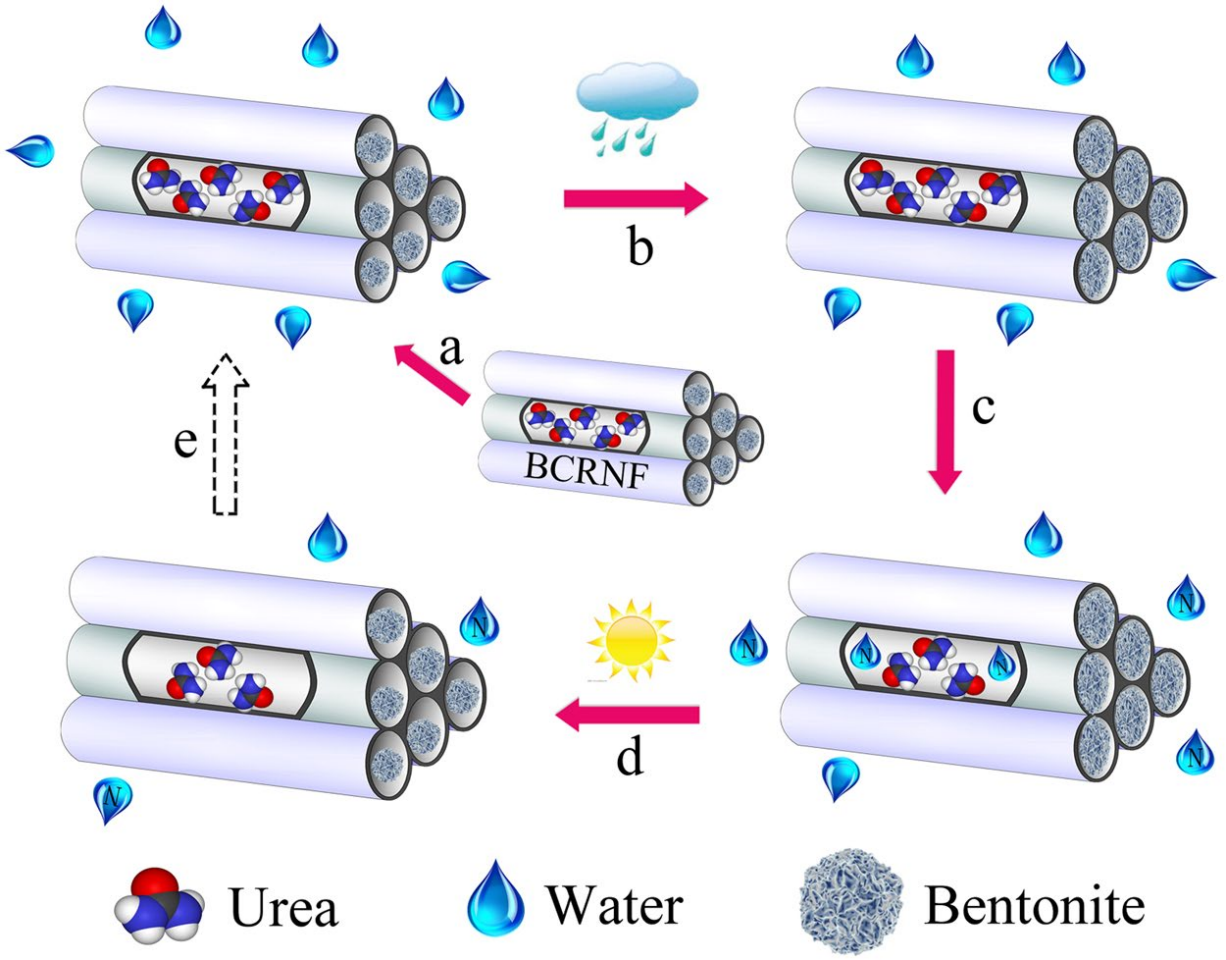
Controlled release mechanism of BCRNF in soil. (**a**) BCRNF in touched with moisture. (**b**) The granules start to absorb moisture and bentonite swells with irrigation water. (**c**) Nutrients dissolve and release slowly through diffusion. (**d**) Bentonite dehydrates and the stored nutrient solution diffuses into soil when there is water shortage. (**e**) BCRNF could absorb moisture when the osmotic pressure is built-up again.

Therefore, it is necessary to discuss the controlled-release mechanism of BCRNF. A multi-stage diffusion model of controlled release mechanism has been proposed to describe the nutrient release^51^. However, the nutrient release from BCRNF incorporates adsorption of biochar and bentonite into the multi-stage diffusion is a distinctive controlled-release process. As shown in Fig. 8, the granules start to absorb moisture, resulting in the swelling of bentonite. Particularly, the swelling of bentonite at the orifice of pores and channels in the biochar can close the permeability channel to prevent nutrient dissolution. Subsequently, osmotic pressure builds up and irrigation water penetrates bentonite and the channel shell of biochar to condense on the solid fertilizer, followed by partial nutrient dissolution. Most irrigation water is absorbed by biochar and part of the nutrient solution is stored in the pores and channels of biochar. Then, the dissolved nutrients are released slowly via diffusion, under concentration or pressure gradient, or a combination of these as the driving force, thereof referred as the “diffusion mechanism.” When water shortage occurs in soil, the stored nutrient solution and water can diffuse to the soil following the dehydration of bentonite. With changes in the osmotic pressure of the soil, some processes including absorption, adsorption, swelling, diffusion, and dehydration of bentonite and biochar take place in parallel and series. In these processes, the bentonite works as a controller: the diffusion and penetration of water and nutrient solution are controlled by the swelling and shrinkage of bentonite. In the last stage, the C-NH-C and CH_2_-CO-NH complex of urea, biochar, and bentonite progressively degrades with a durable and slow release, because van der Waals bonding and π-π electron donor-acceptor interactions disappear depending on the change of environmental pH, ion exchange, microorganism and other factors. The degradation of C-NH-C and CH_2_-CO-NH complex can resist higher temperatures with greater moisture content.

## Conclusions

BCRNF were prepared via the incorporation of urea and bentonite into biochar through hydrothermal synthesis. This synthesis led to interactions among biochar, urea, and bentonite, thus contributing to water retention and controlled release properties of the final products. The structure and properties of BCRNFs were characterized by XRD, FTIR, XPS, SSA, and SEM. Urea particles filled the inner pores and channels of biochar and a new organic complex was generated. The cumulative release amounts of N is 61.3% within 28 days when incubated in water and 54.6% within 98 days when incubated in soil, demonstrating favourable controlled-release properties of the BCRNF. When added to soil, BCRNF can improve the WH% and WR% of the soil because bentonite and biochar absorb water. We believe that this work opens up the potential application of BCRNF produced by hydrothermal synthesis for sustainable agriculture and green revolution.

## Materials and Methods

### Biochar production

Biochars were derived from oilseed rape straws collected from the field station at Hunan Agricultural University, Southern China. Oilseed rape straws were air-dried, and then ground into particles with size smaller than 0.3 mm. Straws were pyrolyzed at 400 °C for 3 h in a laboratory-scale pyrolysis unit under a 3 L·min^−1^ N_2_ flow. The pyrolysis unit comprises a tube reactor equipped with a programmable temperature controller. All preparations were carried out in duplicate.

### Preparation of BCRNF

A solution containing 2 g of urea, and 100 ml distilled water was introduced into a 250 mL sealed reactor containing 40 g of biochar at 135 °C for 2 h (as precursors for BCRNF). A partial dose of the precursor mixture was dried, and the material is referred to as BNF-2). After the reactor was cooled to room temperature, 3 g of bentonite and 2 g of PVA were mixed with the precursor. Then, the sealed reactor was transferred to an oven at 135 °C and heated for 4 h. The resulting product was dried to a constant weight in an oven at 70 °C, and stored for future use. The obtained product is referred to as BCRNF.

### Preparation of control fertilizers

To compare the controlled release effect, we prepared other biochar-based urea fertilizers. A solution containing 2 g of urea and 100 mL distilled water was introduced into a 250 mL sealed reactor containing 2 g of biochar at 135 °C for 2 h. After it was cooled to room temperature, 1 g of bentonite and 1 g of PVA were added to the mixture in the sealed reactor. Then, the sealed reactor was transferred to an oven at 135 °C and heated for 4 h; the dried product is referred to as BCRNF-1.

Using the same production process, 2 g of urea, 21 g of biochar, 2 g of bentonite, and 1 g of PVA were sequentially added to the sealed reactor; the obtained product is referred to as BCRNF-2.

Further, 4 g of urea and 100 mL distilled water were added to a 250 mL reactor containing 20 g of biochar at 135 °C and reacted for 4 h, the product obtained after drying is referred to as BNF-4.

A mixed solution containing 2 g of urea, 2 g of bentonite, and 100 mL distilled water was introduced into a 250 mL reactor containing 40 g of biochar, and then the mixture was dried; the obtained product is referred to as BBNF.

### Optimizing experimental design using the surface response model

The response surface method can solve multivariate problems to fit the functional relationship between the factor and the response value using a multiple quadratic regression equation. Box-benhnken design in the Design-Expert V8.0.6 software was employed to design the surface response model. The amounts of biochar, bentonite and PVC were selected as the design variables. Based on preliminary tests, the amount of urea was set at 2 g, and the amounts of biochar, bentonite, and PVA were set at 2–40 g, 1–3 g, and 0–2 g, respectively. The test design is shown in Table S3), Cumulative nitrogen release rate over 28 days was set as the response value.

### Characterization of materials

The pH of Biochar was determined at a solid-to-liquid ratio of 1:20 (w/v) after 18 h. A CHN/O elemental analyzer (PerkinElmer 2400 Series II, USA) was used to determine after C, H, and N contents of biochar. The total N contents of the samples were measured using H_2_SO_4_–H_2_O_2_ digestion via the Kjeldahl procedure^52^. XRD was conducted using a Bruker D8 X-ray diffractometer (Cu Kα, 40 kV, 20 mA, λ = 1.54056 Å) to characterize any crystallographic structure in biochar. Surface functional groups and bonding modes of organic molecules were determined by FTIR spectroscopy (Thermo-Nicolet Nexus 670, USA). XPS was carried out to analyze surface composition in ~10 nm depth of biochar using an ESCALAB 250Xi spectrometer (THERMO FISHER, USA) with a focused monochromatized Al Kα radiation (hν = 1486.6 eV). Biochar surface morphology was observed by SEM (Zeiss Sigma) with EDS (Bruker) at a working distance of 8–9 mm and an acceleration voltage of 10–15 kV. N_2_ adsorption was conducted on a 3H-2000PS2 Analyzer to identify the SSA and pore volume (PV) of samples. Before measuring the SSA, the samples were pretreated to eliminate other adsorbed substances from the surface (degassing) by heating them in vacuum at 105 °C for 12 h. The total internal SSA of the samples was calculated from the nitrogen adsorption isotherm through Brunauer−Emmett−Teller analysis^53^. The density functional theory method was used to characterized the pore size distribution from the adsorption isotherm, particularly, the method of quenched solid-state functional theory was employed to consider slit/cylindrical pores^54^.

### Controlled-release behaviour of BNF, BBNF, and BCRNF in water and soil

To evaluate nitrogen release, 1 g of each of BNFs, BBNFs and BCRNFs was placed in separate dialysis membrane bags (molecular weight cutoff = 12−14 KDa). The dialysis bags were immersed in 100 mL distilled water and incubated at 20 °C in a water bath. At intervals (1, 3, 7, 14, 21, 28, 42 days or the time until the cumulative N release of fertilizers reached over 80%), 5.0 mL of the solution outside each dialysis bag was collected, then 5.0 mL of fresh distilled water was added to keep the total liquid volume at 100 mL^20,22^. The total N concentration of the collected solution was measured after H_2_SO_4_–H_2_O_2_ digestion via the Kjeldahl procedure^52^.

To evaluate the controlled-release behaviour of BNF, BBNF and BCRNF in soil, samples with nitrogen content equivalent of 1 g urea were sealed in non-woven plastic mesh bags. Then, each bag was buried 5 cm beneath the surface of the soil in a pot containing 200 g of dry soil (below 20-mesh) at 20 ± 2 °C^22^. Soils for these experiments were obtained from the Hunan Agricultural University field station and had physical and chemical properties shown in Table S4 in Supporting Information. The soil had silt loam texture and it consisted of 61.89% silt, 21.84% sand, and 16.27% clay. During these experiments, the WH% of the soil was maintained at 40% by weighing and adding distilled water when necessary. A control treatment involving untreated urea was also included. After 1, 7, 14, 28, 42, 56, 70, and 98 days, the mesh bags were retrieved and dried at room temperature, the remaining N-nutrient content of the residual fertilizer was determined by the Kjeldahl procedure. Each treatment was performed in triplicate.

### Measurement of WH% and WH% capacity of soil with BCRNFs

These experiments were conducted at 20 ± 2 °C at a relative humidity of 40–50%. WH% was measured under three different treatment conditions: 200 g of dry soil only (Control), 200 g of dry soil mixed with 2.0 g of the samples (BNF-2, BBNF or BCRNF). Each sample was placed in a polyvinyl chloride tube with 4.5 cm diameter, two layers of nylon fabric (200-mesh) were placed at the bottoms of the tubes and weighed (marked W0). Each treatment was performed in triplicate. From the top of the tube, the soil samples were slowly infused with distilled water until it seeped. When no leachate was found at the tube bottom, the tube was weighed again to mark W1. The WH% of the soil was calculated using Eq. (1) ^22,55^:

1 $${\rm{WH}} \% =\frac{{{\rm{W}}}_{1}-{{\rm{W}}}_{0}}{200}\times 100 \% $$

After being tested for the WH%, the tubes with samples were kept under identical conditions at 20 ± 2 °C and weighed every 3 days (Wi) for 30 days. The WR% of the soil with BNFs-2, BBNFs or BCRNFs was calculated using Eq. (2) ^22,55^:

2 $${\rm{WH}} \% =\frac{{{\rm{W}}}_{{\rm{i}}}-{{\rm{W}}}_{0}}{{{\rm{W}}}_{1}-{{\rm{W}}}_{0}}\times 100 \% $$

## Supplementary information

Supporting Information for A Biochar-Based Route for Environmentally Friendly Controlled Release of Nitrogen: Urea-Loaded Biochar and Bentonite Composite
