# Supplementary material for: A Biochar-Based Route for Environmentally Friendly Controlled Release of Nitrogen: Urea-Loaded Biochar and Bentonite Composite
Source: Sci Rep. 2019 Jul 2;9:9548. doi: 10.1038/s41598-019-46065-3 (PMC6606633; doi:10.1038/s41598-019-46065-3)
Supplement: Supplementary file 1 — Supporting Information for A Biochar-Based Route for Environmentally Friendly Controlled Release of Nitrogen: Urea-Loaded Biochar and Bentonite Composite [file 41598_2019_46065_MOESM1_ESM.docx]

**Supporting Information**

**A Biochar-Based Route for Environmentally Friendly Controlled Release of Nitrogen: Urea-Loaded Biochar and Bentonite Composite**

Xiangrong Liu^†, ‡, §, #^, Jiayuan Liao ^†, §, #^, Haixing Song^†, §^, Yong Yang^†, §^, Chunyun Guan ^†^, Zhenhua Zhang *^, †, §^

^†^*Southern Regional Collaborative Innovation Center for Grain and Oil Crops in China, College of Resources and Environmental Sciences, Hunan Agricultural University, Changsha 410128, China*

^‡^ *Hunan Qidong Country* *Tobacco Company, Hengyang, 421600, China*

^§^ *National Engineering Laboratory on Soil and Fertilizer Resources Efficient Utilization, Changsha 410128, China*

^#^ *These authors contributed equally to this work.*

*^*^ E-mail: zhzh1468@163.com*

**Contents**

Table S1. The physicochemical properties of samples.

Table S2. Relative ratio of peaks in C1s, N1s and O1s from XPS of biochar and BCRNFs.

Table S3. Factors and levels in three-factor and three-level response surface analysis.

Table S4. Physical and chemical properties of the selected soil.

Figure S1. X-ray diffraction patterns of urea (a), BNF-2 (b), BNF-4 (c), BBNF (d), biochar (e), and BCRNF (f).

Figure S2. SEM elemental mapping of C, N, O, Al, Si, P, Cl and K for the sectioned biochar.

Figure S3. SEM elemental mapping of C, N, O, Al, Si, P, Cl and K for the sectioned BNFs-2.

Figure S4. SEM elemental mapping of C, N, O, Al, Si, P, Cl and K for the sectioned BCRNFs.

Figure S5. Pore size distribution corresponding to SSA of Biochar, BNFs-2, and BCRNFs.

Figure S6. Water-holding (a) and water-retention (b) capacities of soil: 200 g of soil, 200 g of soil with 2 g of BNF-2, 200 g of soil with 2 g of BBNF, and 200 g of soil with 2 g of BCRNF.

**RESULTS**

Table S1. The physicochemical properties of samples.

| Samples | pH | Ash contents (%) | Element analysis (%) | | | N (g/kg) | K (g/kg) | P  (g/kg) | Porosity parameters | | |
| --- | --- | --- | --- | --- | --- | --- | --- | --- | --- | --- | --- |
|  |  |  | C | H | O |  |  |  | SSA_BET_ (m^2^/g) | V_T_ (m^3^/g) | D_p_ (nm) |
| Biochar | 9.84 | 9.6 | 61.5 | 4.2 | 25.6 | 1.24 | 3.68 | 0.32 | 23.84 | 0.015 | 2.58 |
| BNF-2 | 7.38 | 9.5 | 61.1 | 4.1 | 25.1 | 42.5 | 3.43 | 0.30 | 1.56 | 0.003 | 2.16 |
| BNF-4 | 6.86 | 9.3 | 59.6 | 4.1 | 24.8 | 75.1 | 3.31 | 0.29 | 1.23 | 0.002 | 2.15 |
| BBNF | 8.47 | 16.8 | 55.8 | 3.7 | 22.9 | 39.7 | 2.74 | 0.22 | 1.33 | 0.002 | 2.31 |
| BCRNF | 9.21 | 18.7 | 53.2 | 3.9 | 24.2 | 26.6 | 3.36 | 0.29 | 1.63 | 0.002 | 2.12 |
| BCRNF-1 | 8.46 | 22.8 | 49.3 | 3.5 | 24.3 | 45.8 | 1.85 | 0.17 | 0.51 | 0.001 | 2.03 |
| BCRNF-2 | 8.15 | 15.3 | 57.4 | 3.8 | 23.4 | 38.2 | 2.65 | 0.20 | 0.62 | 0.001 | 2.05 |

Table S2. Relative ratio of peaks in C1s, N1s and O1s from XPS of biochar and BCRNF.

| Samples | C1s | | | | | N1s | | | O1s | | | |
| --- | --- | --- | --- | --- | --- | --- | --- | --- | --- | --- | --- | --- |
|  | C–C | C=C | C–O–R | C–R | COOR | N–C | N–H | (C)_3_–N | C–OH | COOR | C=O | SiO_2_ |
| Biochar | 14.3 | 34.8 | 21.6 | 15.8 | 13.5 | 59.7 | 40.3 | / | 40.4 | 26.5 | 33.1 | / |
| BCRNF | 15.3 | 32.6 | 23.6 | 14.8 | 13.7 | 33.1 | 59.1 | 8.8 | / | 33.9 | 35.6 | 30.5 |


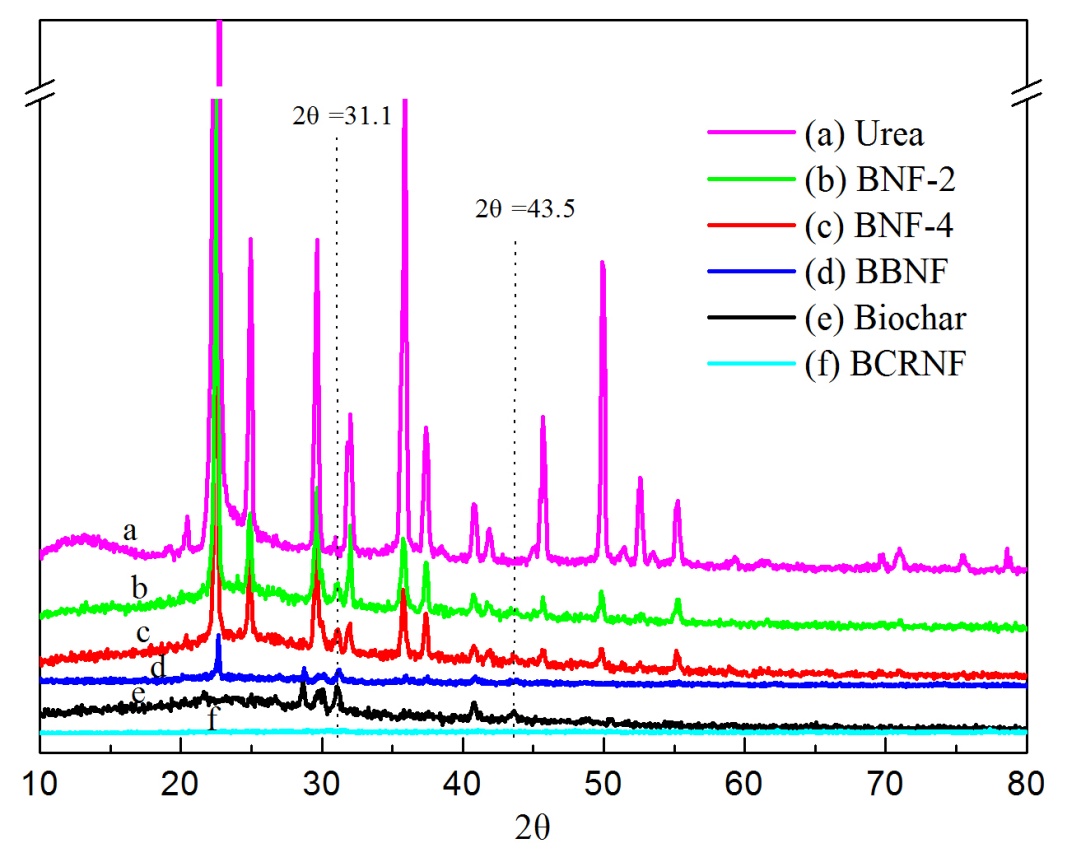


Figure S1. X-ray diffraction patterns of urea (a), BNF-2 (b), BNF-4 (c), BBNF (d), biochar (e), BCRNF (f).


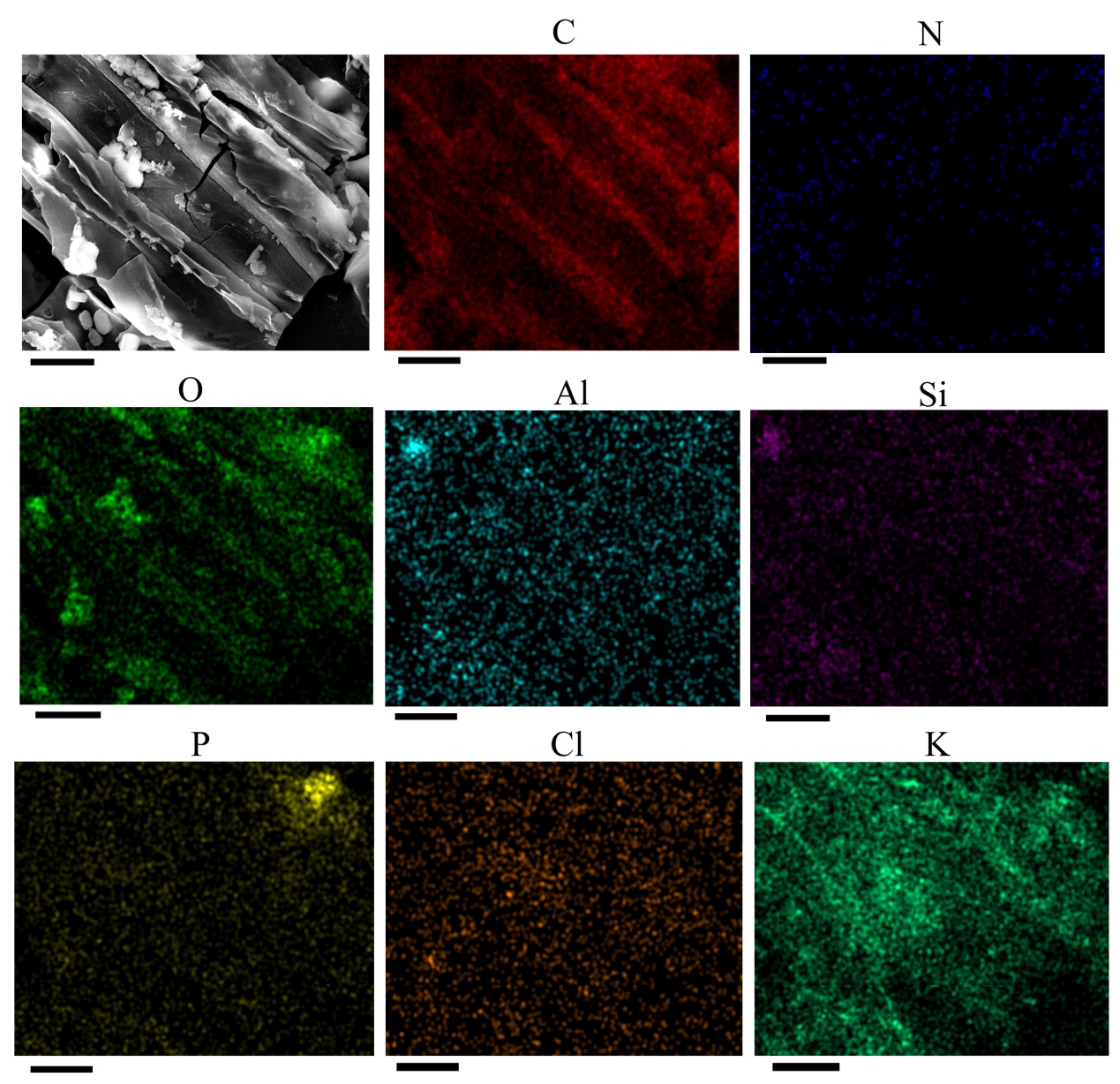


Figure S2. SEM elemental mapping C, N, O, Al, Si, P, Cl and K for the sectioned biochar (scale bar, 5 µm).


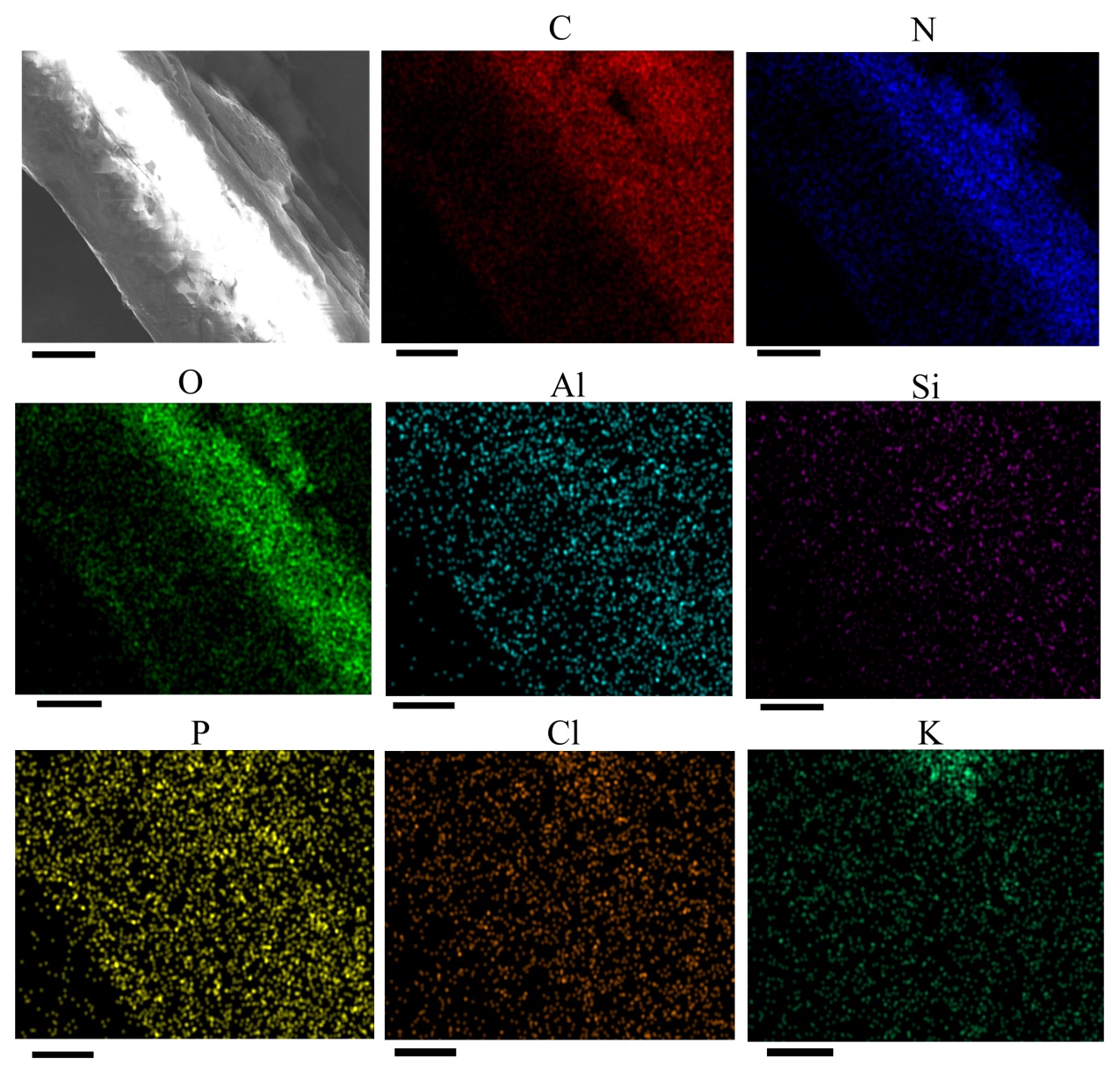


Figure S3. SEM elemental mapping C, N, O, Al, Si, P, Cl and K for the sectioned BNFs-2 (scale bar, 2.5 µm).


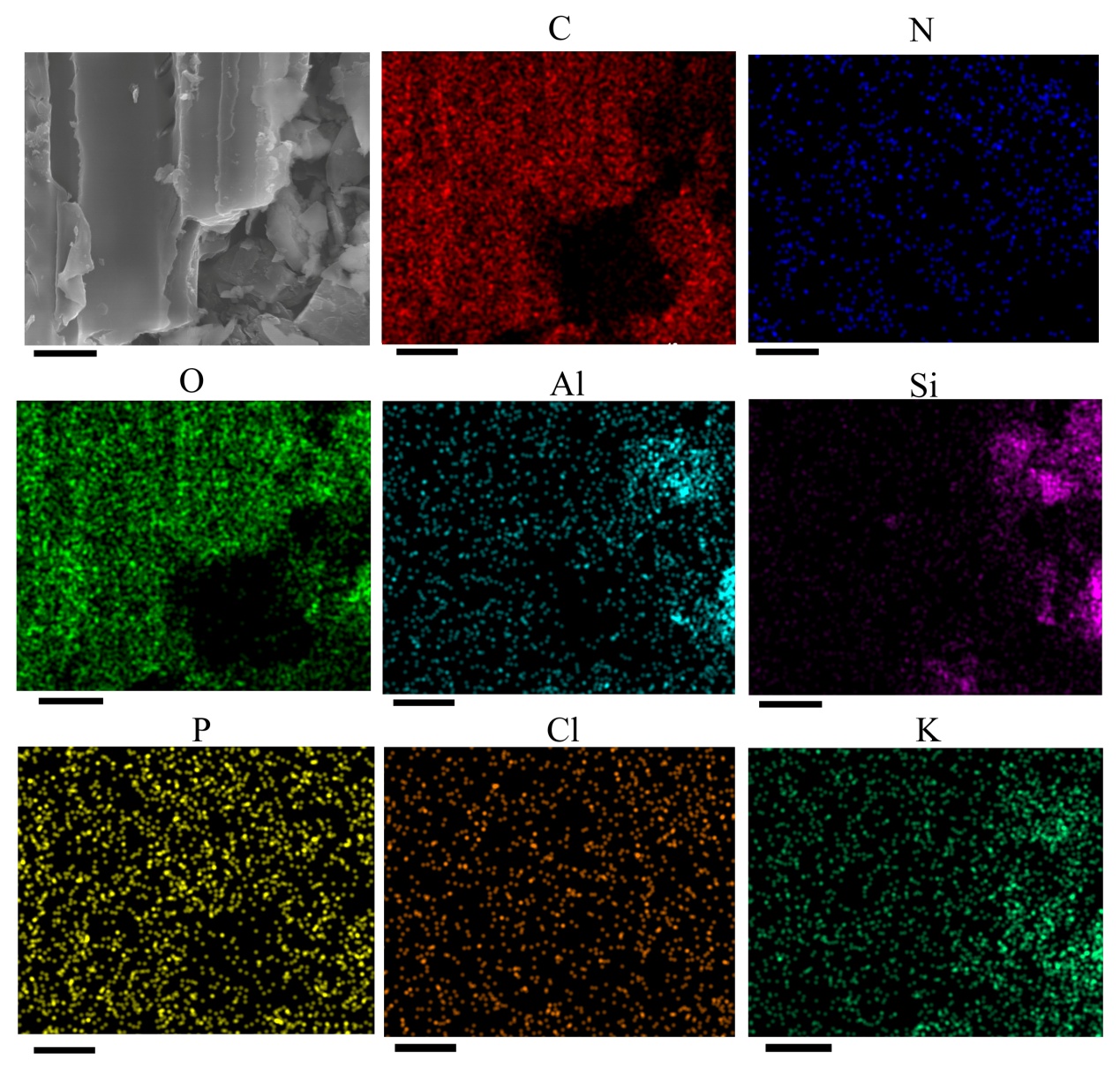


Figure S4. SEM elemental mapping C, N, O, Al, Si, P, Cl and K for the sectioned BCRNFs (scale bar, 2.5 µm).

The SEM image and elemental mapping sequence of the selected regions of biochar, BNF-2, BCRNF were shown in Figure S2, Figure S3, Figure S4, respectively. In Figure S1, biochar was rich in carbon and oxygen, traces of N element. Compared with biochar and BNF-2, the N content of BCRNF is higher than biochar, and lower than BNF-2, indicated that urea was filled into the pores of biochar and PVA covered with the particle. The Si and Al rich regions were observed in the orifice of pores and channels of the biochar in Figure S4, this result was in accordance with the SEM morphology of BCRNF.


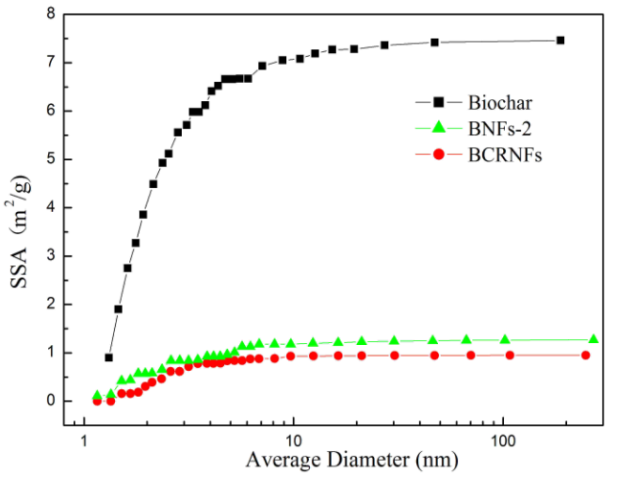


Figure S5. Pore size distribution corresponding to SSA of Biochar, BNFs-2, and BCRNFs.


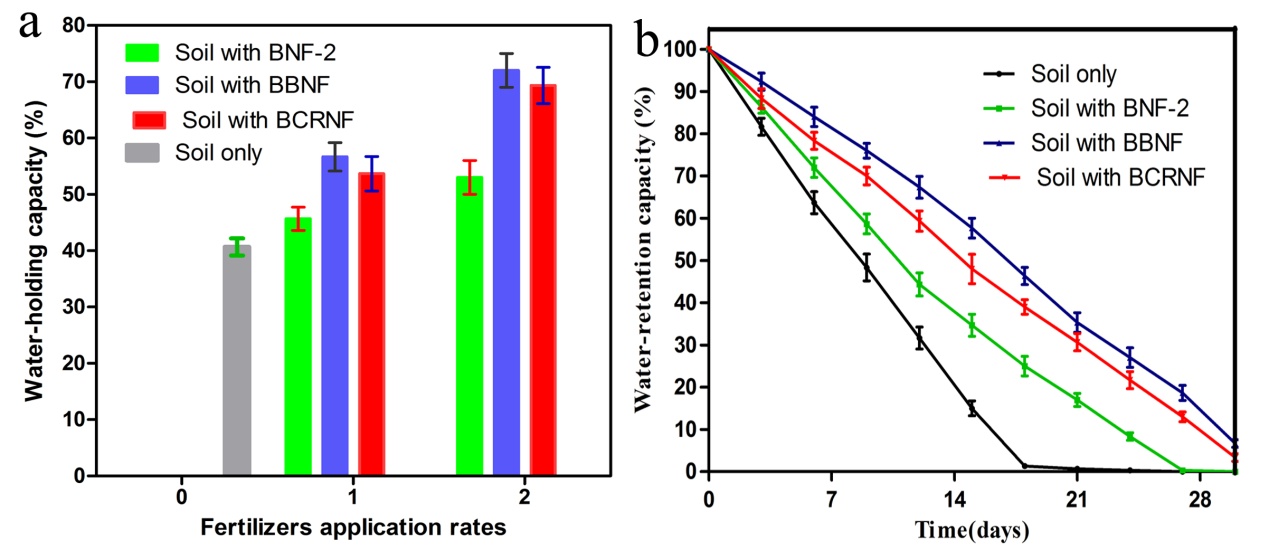


Figure S6. Water-holding (a) and water-retention (b) capacities of soil: 200 g of soil, 200 g of soil with 2 g of BNF-2, 200 g of soil with 2 g of BBNF, and 200 g of soil with 2 g of BCRNF.

**Discussion.**

**Temperature of biomass pyrolysis**

The retention of nutrients by biochar could be affected by biochar pyrolysis temperature, soil types, fertilizer doses, and soil water content. During biomass pyrolysis, a large number of reactions take place in parallel and series, including dehydration, depolymerization, isomerization, aromatization, decarboxylation, and charring[^1^](#_ENREF_1)^,^[^2^](#_ENREF_2). For most biochars from which feedstock types of biomass are typically generated, decomposition generally occurs during the primary decomposition to form solid char at 200–400 °C, which is responsible for 95% of total degradation of biomass[^3^](#_ENREF_3). It is widely accepted that primary decomposition of biomass material (<400 °C) consists of a degradative process, whereas the secondary thermolysis (>400 °C) proceeds within the solid matrix and involves an aromatization process[^3^](#_ENREF_3). The degradation pathways of the main biomass components (cellulose, hemicelluloses, and lignin) have been investigated separately. Cellulose decomposition primarily occurs between 325 and 400 °C and levoglucosan is the main pyrolysis product[^4^](#_ENREF_4). Decomposition of hemicelluloses mainly occur between 250 and 350 °C with rapid depolymerization of the main chain (xylopyranose chain in the case of xylan and glucopyranose–mannopyranose chain in the case of glucomannan) occurring at around 290 °C for xylan and 310 °C for glucomannan^[4](#_ENREF_4)^. Lignin with its high content of benzene rings is the most stable component and as such decomposes at higher temperatures of 300–550 °C with the highest decomposition rate generally comprised between 360 and 400 °C[^5^](#_ENREF_5). In addition, nutrient contents of biochar can be determined by feedstock source and pyrolytic temperature. N losses begin at about 400 °C, with half of the N content lost as volatiles at about 750 °C in woody and herbaceous biochars^[6](#_ENREF_6)^. The contents of available N (water-soluble) in biochars decreased from 39 to 8mg kg^−1^ with increase in pyrolysis temperatures from 350 to 600 °C, which could be attributed to the loss of total N and the heterocyclization of N during pyrolysis^[7](#_ENREF_7)^. In addition, the reduced N_2_O emissions is attributed to the content of polycyclic aromatic hydrocarbons in low-temperature biochars (300–400 °C), but not in the high-temperature biochars (>500 °C), because biochars produced at 200 °C contain a relatively large amount of phenolic compounds and resulted in markedly reduced N_2_O emission[^8^](#_ENREF_8). Biochar treated at a high temperature (>600 °C) shows reduced NH_4_^+^-N uptake by physisorption and NO_3_^−^-N by chemisorption[^9^](#_ENREF_9)^,^[^10^](#_ENREF_10). Soil NH_4_^+^-N was decreased with biochars processed at low and high temperatures (<400 and >600 °C, respectively)[^9^](#_ENREF_9). Moreover, the soil NO_3_^−^-N was decreased by biochar after low temperature treatment (<400 °C)^[9](#_ENREF_9)^. According to above literatures and analysis, it is important that the appropriate pyrolysis temperature of biomass is 400 °C to obtain higher yield and quality of biochar products with high nutrient retention properties.

**EXPERIMENTAL SECTION**

**Materials.**

Urea, Bentonite and PVA were purchased from Shanghai Macklin Biochemical Co., Ltd. Bentonite had a composition of Al_2_O_3_·4SiO_2_·H_2_O. Alcoholysis degree of PVA is 88 mol%. ^15^N-Urea (Shanghai Research Institute of Chemical Industry, China) was applied to determine the distribution of N in grain and plant.

**Sample analysis of soil**

For the soil samples, pH was determined in a 1:5 (w/v) soil to water slurry using a pH-meter (AB150, Fisher Scientific, USA). SOM was determined using an oxidation method with potassium dichromate.[^11^](#_ENREF_11) Total N content of soil samples was determined using an automatic azotometer (KDN-102F, Qianjian Ltd., Shanghai).[^12^](#_ENREF_12) Total P was measured by sodium hydroxide fusion, followed by colorimetric analysis. Olsen-P (extracted with 0.5 M NaHCO_3_) were determined by a segmented continuous flow analyzer (Quaatro, Bran+Luebbe, Germany).Total K was measured by flame photometry after sodium hydroxide fusion, and the available K was extracted with NH_4_OAc and determined by flame photometry.

Table S3. Factors and levels in three-factor and three-level response surface analysis.

| **No.** | **Amount of Biochar (g)**  **A** | **Amount of bentonite (g)**  **B** | **Amount of PVA (g)**  **C** |
| --- | --- | --- | --- |
| 1 | 21.00 | 2.00 | 1.00 |
| 2 | 21.00 | 2.00 | 1.00 |
| 3 | 40.00 | 2.00 | 0.00 |
| 4 | 2.00 | 3.00 | 1.00 |
| 5 | 2.00 | 1.00 | 1.00 |
| 6 | 21.00 | 2.00 | 1.00 |
| 7 | 40.00 | 1.00 | 1.00 |
| 8 | 21.00 | 1.00 | 0.00 |
| 9 | 21.00 | 3.00 | 2.00 |
| 10 | 2.00 | 2.00 | 2.00 |
| 11 | 21.00 | 1.00 | 2.00 |
| 12 | 21.00 | 2.00 | 1.00 |
| 13 | 21.00 | 3.00 | 0.00 |
| 14 | 21.00 | 2.00 | 1.00 |
| 15 | 40.00 | 3.00 | 1.00 |
| 16 | 40.00 | 2.00 | 2.00 |
| 17 | 2.00 | 2.00 | 0.00 |

Table S4. Physical and chemical properties of the selected soil.

| pH  value | SOM content  (g·kg^-1^) | TN content  (g·kg^-1^) | TP content  (g·kg^-1^) | TK content  (g·kg^-1^) | AP content  (mg·kg^-1^) | AK content  (mg·kg^-1^) | NO_3_^-^-Ncontent  (mg·kg^-1^) | NH_4_^+^-Ncontent  (mg·kg^-1^) |
| --- | --- | --- | --- | --- | --- | --- | --- | --- |
| 5.55 | 15.19 | 1.52 | 0.51 | 5.52 | 18.87 | 81.42 | 36.56 | 20.15 |

References

1 Collard, F. X. & Blin, J. A review on pyrolysis of biomass constituents: Mechanisms and composition of the products obtained from the conversion of cellulose, hemicelluloses and lignin. *Renew Sust Energ Rev* **38**, 594-608 (2014).

2 Kan, T., Strezov, V. & Evans, T. J. Lignocellulosic Biomass Pyrolysis: A Review of Product Properties and Effects of Pyrolysis Parameters. *Renew Sust Energ Rev* **57**, 1126-1140 (2016).

3 Fisher, T., Hajaligol, M., Waymack, B. & Kellogg, D. Pyrolysis behavior and kinetics of biomass derived materials. *Journal of Analytical & Applied Pyrolysis* **62**, 331-349 (2002).

4 Stefanidis, S. D. *et al.* A study of lignocellulosic biomass pyrolysis via the pyrolysis of cellulose, hemicellulose and lignin. *Journal of Analytical & Applied Pyrolysis* **105**, 143-150 (2014).

5 Wang, S. *et al.* Comparison of the pyrolysis behavior of lignins from different tree species. *Biotechnol Adv* **27**, 562-567 (2009).

6 Lang, T., And, A. D. J. & Jensen, P. A. Retention of Organic Elements during Solid Fuel Pyrolysis with Emphasis on the Peculiar Behavior of Nitrogen. *Energ Fuel* **19**, 1631-1643 (2005).

7 Zheng, H. *et al.* Characteristics and nutrient values of biochars produced from giant reed at different temperatures. *Bioresource Technol* **130**, 463 (2013).

8 Wang, Z. *et al.* Characterization and influence of biochars on nitrous oxide emission from agricultural soil. *Environ Pollut* **174**, 289-296 (2013).

9 Nguyen, T. T. N. *et al.* Effects of biochar on soil available inorganic nitrogen: A review and meta-analysis. *Geoderma* **288**, 79-96 (2017).

10 Kameyama, K., Miyamoto, T., Shiono, T. & Shinogi, Y. Influence of sugarcane bagasse-derived biochar application on nitrate leaching in calcaric dark red soil. *J Environ Qual* **41**, 1131 (2012).

11 Sciubba, L., Cavani, L., Marzadori, C. & Ciavatta, C. Effect of biosolids from municipal sewage sludge composted with rice husk on soil functionality. *Biology & Fertility of Soils* **49**, 597-608 (2013).

12 Bai, J. H. *et al.* Spatial and temporal distribution patterns of nitrogen in marsh soils from an inland alkaline wetland – A case study of Fulaowenpao wetland, China. *Acta Ecologica Sinica* **30**, 210-215 (2010).
